# Distributed decision making in action: diagnostic imaging investigations within the bigger picture

**DOI:** 10.1002/jmrs.250

**Published:** 2017-11-11

**Authors:** Chandra R. Makanjee, Anne‐Marie Bergh, Willem A. Hoffmann

**Affiliations:** ^1^ University of Canberra Canberra Australian Capital Territory Australia; ^2^ South African Medical Research Council Unit for Maternal and Infant Health Care Strategies University of Pretoria Pretoria South Africa; ^3^ Tshwane University of Technology Pretoria South Africa

**Keywords:** Diagnostic imaging, distributed decision making, radiography, South Africa

## Abstract

**Introduction:**

Decision making in the health care system – specifically with regard to diagnostic imaging investigations – occurs at multiple levels. Professional role players from various backgrounds are involved in making these decisions, from the point of referral to the outcomes of the imaging investigation. The aim of this study was to map the decision‐making processes and pathways involved when patients are referred for diagnostic imaging investigations and to explore distributed decision‐making events at the points of contact with patients within a health care system.

**Method:**

A two‐phased qualitative study was conducted in an academic public health complex with the district hospital as entry point. The first phase included case studies of 24 conveniently selected patients, and the second phase involved 12 focus group interviews with health care providers. Data analysis was based on Rapley's interpretation of decision making as being distributed across time, situations and actions, and including different role players and technologies.

**Results:**

Clinical decisions incorporating imaging investigations are distributed across the three vital points of contact or decision‐making events, namely the initial patient consultation, the diagnostic imaging investigation and the post‐investigation consultation. Each of these decision‐making events is made up of a sequence of discrete decision‐making moments based on the transfer of retrospective, current and prospective information and its transformation into knowledge.

**Conclusion:**

This paper contributes to the understanding of the microstructural processes (the ‘when’ and ‘where’) involved in the distribution of decisions related to imaging investigations. It also highlights the interdependency in decision‐making events of medical and non‐medical providers within a single medical encounter.

## Introduction

Decision making involves choosing a course of action to achieve specific outcomes.^1^ Within the health care system decision making occurs at multiple levels, including the organisational, departmental and individual levels. In the medical literature decision making is extensively explored from different viewpoints using terms such as ‘informed’,^2^ ‘shared’,^3^, ^4^, ^5^ ‘collaborative’,^6^ ‘participatory’, ‘paternal’^7^ and ‘negotiated’ decisions.^8^ Within the ambit of diagnostic imaging, van Baalen et al.^9^, in their study on the diagnosis and treatment of patients with pulmonary hypertension, refer to this kind of decision making as being based on ‘distributed knowing’. Distributed knowing implies a socially distributed process of shared meaning making among different health care providers. Information is exchanged, collectively explored and adjusted at the patient's different points of contact in a medical encounter. Within the health care context the ultimate predictor of the efficiency and effectiveness of the decision is measured by the well‐being of the patient, hence the prominence given to patient‐centred care.

Diagnostic tests are important informative tools in aiding medical practitioners to make a final diagnosis or draft a treatment plan. A referral for a diagnostic imaging investigation forms part of an intermediate and/or preliminary action, namely a decision to request a radiological opinion to detect or exclude disease. This radiological opinion is formed on the basis of the radiographic component of the action or event that ensures the provision of high‐quality images.^10^, ^11^ In other words, from a clinical decision‐making point of view the outcomes of an imaging investigation contribute to the intermediate goal of determining the prognosis, which may in turn prompt a reconsideration or reformulation of the goals of the treatment and management plan, the monitoring of the clinical course of disease and/or the screening for at‐risk health.^12^

Diagnostic imaging investigations may take place in different intra‐ and inter‐institutional locations. They involve several role players in the context of referral for and performance of diagnostic investigations. Therefore, relations between multi‐professional providers and between providers and patients are in constant flux. On the one hand, diagnostic imaging is ‘a highly structured, production‐oriented, complex, distributed, technical and image‐centred activity’ (p. e12).^13^ On the other hand, multiple human interactions^14^ and high levels of interdependency between professional groups have a bearing on individual imaging decisions^15^ related to matters such as the adequacy of information on the request form, the most justifiable investigation with the most appropriate radiographic technique and/or intervention, and methods of image acquisition and processing to obtain images of optimal diagnostic quality.

The aim of this study was to map the decision‐making processes and pathways involved in patient referrals for diagnostic imaging investigations and to explore distributed decision‐making events at patients’ points of contact with the health care system.

## Methods

This exploratory qualitative study was conducted in a South African academic public health complex in 2009 and 2010 providing care from primary to tertiary health care, also in terms of access and availability of diagnostic imaging modalities. The main setting was the district hospital, the patients’ entry point into the system.

The Research Ethics Committee of the Faculty of Health Sciences of the University of Pretoria (reference number: 107/2008) approved this study. All participating institutions granted permission for the study and all potential patient and health care provider participants signed written informed consent before enrolment in the study.

### Data collection

The study was conducted in two phases. The first phase consisted of 24 conveniently selected patient case studies. Each consenting patient was shadowed by the primary researcher from entry into the system at either casualty or the outpatients department of the district hospital to the point of exit from the health care complex. Shadowing necessitated multiple data collection strategies, which included observations at the multiple points of provider–patient contact, patient entry and exit interviews, individual interviews with attending providers, informal conversations with patient and provider participants and document analysis. The details of the data collection methodology have been described elsewhere.^16^ Most of the case study shadowing lasted 1–3 h, with a few lasting 2 weeks (*n *=* *1) or 2 months (*n *=* *2). Most medical consultations, as well as patient and provider interviews, were audio‐recorded and detailed field notes were made of observations and informal interactions.

The findings from the first phase informed the second phase of the study, namely 12 audio‐recorded focus group interviews with different professional provider groups with a view to obtain a more in‐depth perspective on observations during the shadowing process. These groups included medical officers and family physicians (3 groups), radiologists and radiology registrars (2 groups), radiographers (3 groups) and nurses (4 groups). The different data collection strategies (i.e. interviews, observations, patient records and focus group interviews) allowed for triangulation between different data sources.

In the exit interviews, patient participants were probed on their experience of being shadowed and interviewed. This also served as a form of member checking with patient participants.

### Data analysis

Data analysis was done manually, using an inductive approach.^17^ After a patient case had been completed, the audio‐recording was transcribed and observational field notes typed up. These data documents were read and re‐read independently by two of the authors to identify potential categories, subthemes and themes. The individual interpretations were compared for similarities, and interpretation differences were solved through consensus. A reiterative process was followed for subsequent cases until thematic saturation was achieved. A similar process was followed for the focus group interviews. The themes identified in the case studies were then compared with the themes emerging from the focus groups.^16^ These themes were subsequently interpreted in relation to the framework described below.

Member checking on the accuracy and feasibility of interpretations was done with some of the professional participants. Peer debriefing with a radiologist and a family physician assisted in determining the level of consistency and accuracy of the interpretations. The use of verbatim quotations from participants enhances the transferability of the findings.

### Framework for interpreting decision‐making themes

We based our interpretation framework on the work of Rapley,^18^ which focuses on decision making ‘distributed across time, courses of actions, people, situations and technologies’ (p. 430). Decision making is therefore taken ‘beyond the immediate space of a single consultation’ (p. 430). The medical practitioner plays a collaborative role where the responsibility for making decisions is distributed across several medical specialists, non‐medical providers and patients, as well as across multiple encounters. In this paper we focus on the medical encounters of patients, going beyond the dyadic doctor–patient consultation. van Baalen et al.^9^ refer to the social nature of distributed processes over people and settings, ‘[b]ecause pieces of evidence are generated by people with different expertise and interpretation and adjustments take place in interaction between different experts’ (p. 1).

Each encounter takes place across a range of clinical and non‐clinical teams within a relatively short period of time and ‘with a smaller cast of characters’ (p. 433).^18^ Patients’ medical encounters are conceptualised as metaphorical journeys, to which Rapley refers as ‘a patient's trajectory of care’ (p. 440).^18^ In these encounters, retrospective, current and prospective information and knowledge are distributed, transferred and transformed across different health care providers; it is also shared between providers and between provider and patient. Decisions are arrived at through transactional interaction processes among health care providers and between health care providers and the patient. Transactions and decision making at the point of diagnostic imaging are further shaped by and mediated through technology.

## Results

**Figure 1:**
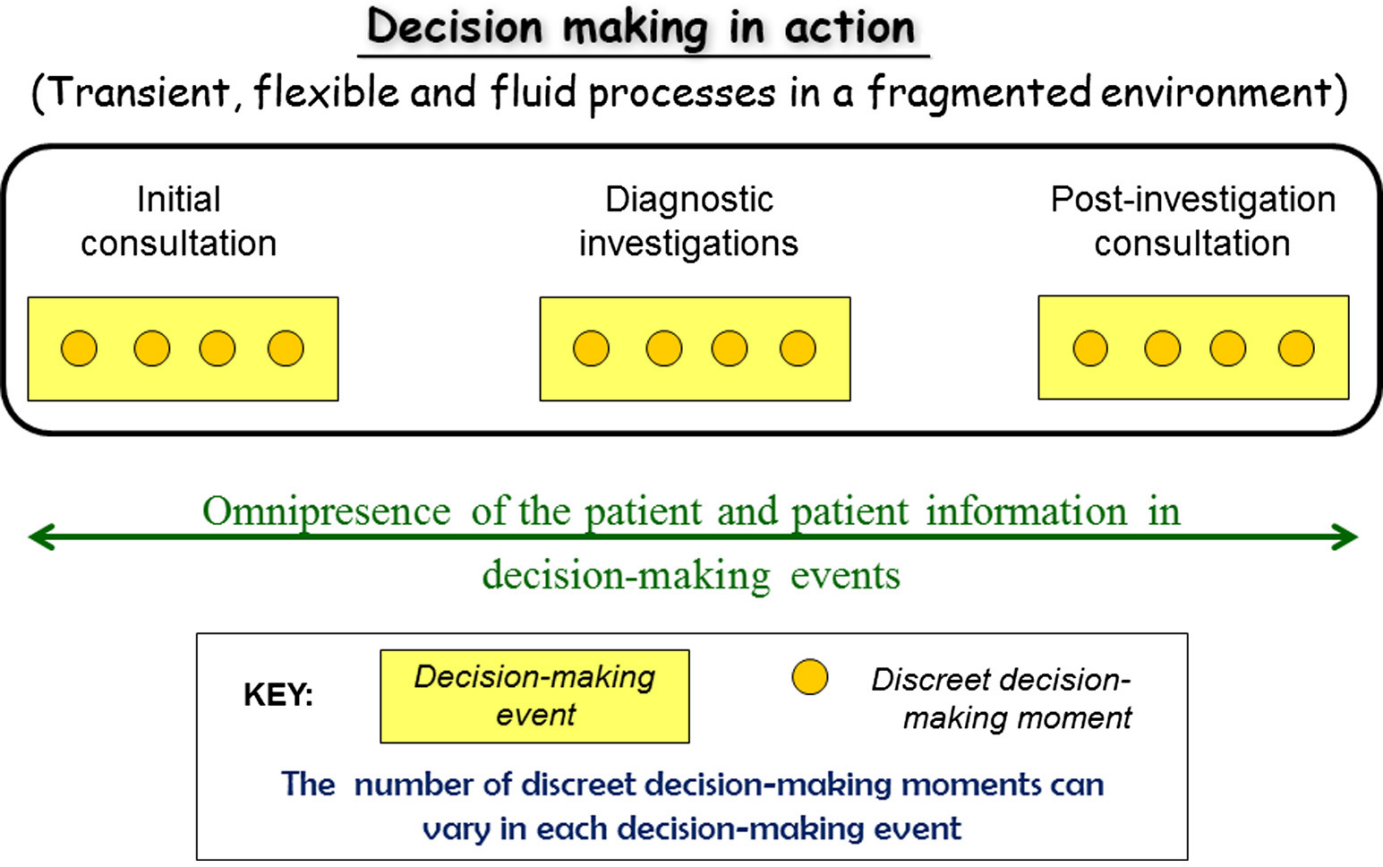
Framework for understanding distributed decision making.

Figure 1 shows the framework that emerged from the analysis of distributed decision making involving diagnostic imaging within the bigger picture of patients’ medical encounters. Clinical decision making within a single medical encounter is distributed across vital points of contact or decision‐making events on a patient's journey. It mainly revolves around the initial patient consultation, the diagnostic imaging investigation and the post‐investigation consultation. The outcomes of the various decision‐making events are integrated and collectively reflected in the final diagnosis and treatment plan. Each of the broader decision‐making events (points of contact) is made up of a sequence of discrete decision‐making moments that can vary in number within a decision‐making event. Each event forms part of the decision making in action that characterises the patient's journey. Health care providers draw on multiple sources of knowledge at each of these points in order to arrive at the final diagnosis and treatment plan. The only person present at all points of contact and ‘present’ at all the decision‐making events is the patient. However, being present does not automatically mean participation in decision making or the presence of patient‐centred care. In our study, patients did not engage in active communication with health care providers and seldom requested a clarification.^19^ Interactions were limited to providers’ requests for information from patients and explanations of their decisions to patients. This is similar to or in contrast to the findings in other studies.^5^

Specific codes are used to denote the various participant groups in the direct quotations. Quotations from the patient cases in phase 1 are denoted with CS and those from health professional focus groups in phase 2 with FG, followed by MP for medical practitioner and RAD for radiographer.

### The initial consultation

In this study, the initial consultation consisted of three main components that broadly correspond to the three types of information and knowledge that are shared and transferred across decision‐making actions. A medical practitioner explained these three components as follows in a focus group:

The patient will be referred with a letter and this is the problem – the doctor suggest[s] you do a mammogram. That is quite a difficult situation**.** You have to explain to the patient, I have to first take the history [retrospective information], examine you [current information] and see if you really need this mammogram [prospective information] because I cannot just send you for a mammogram after examining you. (FGMP2)

#### Retrospective knowledge

The patient's history is taken with a view to retrospectively filling in gaps related to knowledge of the patient and his or her condition (information gathering and clarification). In our study, the process of filling these gaps was sometimes compromised because patient files were missing or lost. Sometimes a medical officer had to attempt to get a full picture of one patient's situation without any previous records*:* ‘So let's just start up fresh, because we don't know where that file is gone’ (CS14).

#### Current knowledge

This is usually gained through physical examination or the use of instrumentation to confirm the retrospective information and to create a basis for further action (interpretation).

#### Prospective knowledge

The information‐gathering and clarification phases are followed by an interpretation phase based on findings from the information‐gathering exercise and the outcomes of the physical assessment. The decision to refer a patient for a diagnostic investigation is taken with a view to possibly gain knowledge that would further inform the diagnosis and treatment plan. A referral decision has also to be made on the type of imaging investigation, the appropriate imaging modality and, where applicable, type(s) of projections.

In most instances where medical officers shared their diagnostic interpretation with patients, a degree of uncertainty or speculation was communicated: ‘It seems to me more of a gastritis food poisoning’ (CS07). The referral decision was also based on a ‘to make sure’ approach (CS01). However, radiographers in the focus groups expressed strong concerns about over‐requesting by medical practitioners:

As they come in we say, ‘What happened to you?’ The patient explains to you, ‘I bumped my knee on the chair or something’. Then the doctor has requested for pelvis, femur, knee and hip. (FGRAD3)

The various decisions in our study were situated and were not always taken in the same order regarding diagnosis, treatment plan and referral for diagnostic imaging investigation. They varied from one medical practitioner to another, and depended on the nature of the presenting problem and the availability of imaging modalities and services. In some cases the medical practitioner formulated a preliminary diagnosis, a possible plan of action and/or potential management strategy before deciding to refer the patient for a diagnostic imaging investigation. A decision at the end of the initial consultation could be either (1) referral of the patient for a further diagnostic opinion or confirmation of a diagnosis (e.g. imaging investigation, laboratory tests or a specialist opinion) or (2) a final decision on the commencement of a management plan (e.g. discharge with treatment and/or referral elsewhere for follow‐up).

### Diagnostic imaging investigations

At the radiography department patients meet a new set of health care providers who carry out certain processes to produce optimal quality images. The three types of information and knowledge are also distributed across decision‐making actions.

#### Retrospective knowledge

For verification and clarification, the radiographer and/or radiologist takes a history and scrutinises the request form for information. In our study, they did not have the in‐depth retrospective knowledge (i.e. patient history) of each patient case: ‘You know [a] little bit about what's going on’ (FGRAD2). Radiographers’ decisions were mostly based on the request forms as primary source of information. According to one senior family medicine consultant, ideally ‘if you are worried about a peptic ulcer that is perforated, you need to write “query peptic ulcer perforation”’ (FGMP3). However, radiographers often encountered inadequate or conflicting referral and/or request information, as illustrated by the following case:

I … read the history [on the request form]… It just state[s] injury. It's quite little to work with …. but most of the time we just read the history and read what they are asking us to do … Then I check with the patient as well because most of the time or sometimes we just need to check with the patient whether it is the left leg because the right leg is in plaster. Do they really want the left leg or [do] they want the right leg? …and sometimes the doctor would write ‘leg’ and a leg is quite a big area. We're not always sure what they want. (CS13)

#### Current knowledge

During the diagnostic imaging, investigation decisions are taken on positioning, projections, exposure technique, image acquisition and processing based on the retrospective knowledge. The current knowledge constitutes the insights gained by the radiographer and/or radiologist about the patient's body and/or region of interest, which may lead to amending decisions regarding additional projections and patient management. The example below illustrates how the absence of retrospectively acquired information can influence the diagnostic imaging investigation.

As it is, I am the first one to see the scans being done. When I pick something up, I would get the doctor's attention right there and then to ask them, ‘Do you think there is something in here that needs further investigation?’… For instance, we were very busy and there was a patient referred from another hospital with cervical spine and a brain scan and the moment I start to scan his brain his brain looked fine. The moment I started scanning his c/spine he had a C1 big fracture and the scan wasn't even done yet. I got the radiologist to look at the scan and immediately she got to the phone to speak to the [referring] doctor … to come – because the patient even came with a soft collar … to the scan – to put on a hard collar on the patient. So … in that respect where you know people just rushing in about trying to get things done and I see something bothering me, which might not necessarily be something. (CS18)

#### Current and prospective knowledge

An evaluation of image quality provides the radiographer and/or radiologist with current and prospective knowledge required to decide whether additional projections are needed and what post‐investigation instructions to give the patient and/or referring doctor. The following is an example of a radiologist's evaluation of hip radiographs of a patient who had been in a motor vehicle accident:

I am not happy with the right hip joint; that's why I would do a further investigation like ultrasound to see if there is an effusion. But then again the clinical, I need to know where the foreign bodies are. Are they superficial or where there is bruising or haematoma in that region? So the hip I would have further investigated. (CS23)

As with the initial consultation, the decision‐making sequence described above was not fixed. For example, verification was sometimes sought from the patient while positioning him or her for a certain projection, especially in the case of an entire upper limb request. The radiographer sought confirmation by asking the patient, ‘It's the right side [arm], okay? What happened?’ (CS01). She then checked with the patient to enable a decision on the region of interest and the most appropriate anatomical structures to include in the projection: *‘*Exactly what is sore? The elbow, wrist or whatever?’ (CS01).

In the course of the encounter at the imaging section, radiographers and radiologists make the decisions on:

1. the quality of the request and whether to accept or reject it, and where needed, whether to contact the colleague on site or the referring practitioner for clarification,
2. patient positioning, comfort and safety and types of projections during the investigation and
3. the quality and completeness of the images and the necessity for repeated or additional projections after the first investigation to address or answer the clinical question at hand.

A further decision could be made by the radiologist as to whether (1) to send a report with recommendations to the referring practitioner, (2) to do follow‐up investigations or (3) to do an investigation with a different modality.

### Post‐investigation consultation

When the medial practitioner sees the patient after the imaging investigation she or he uses current knowledge of the patient's condition provided by the images and/or the radiological report together with the retrospective knowledge to make further decisions regarding the plan of action and management of the patient. Three main types of decisions were reported in our study: (1) discharge of a patient with treatment, (2) admission of a patient to the district hospital for further investigation(s) and (3) treatment at or transfer to a higher level of care.

We will discharge if the infection is not so severe. Depending on the test results we will give you medication. But if it is severe, we are going to keep you here for a day’. (CS05)

An example of a transfer to a higher level of care (3) was a patient referred to surgical outpatients at the tertiary hospital for a mammogram or biopsy, who received the following explanation:

They [practitioner at the district hospital] say: ‘Groups of atypical abnormal epithelial cells, biopsy is recommended.’ The problem is if [I] don't have a picture of a mammogram then where are you going to do that biopsy? … So we want to see you after this x‐ray [mammogram] that they take. You must bring all … [records] with [you], so we don't lose track of what is going on. (CS13)

## Discussion

The study reported in this paper is one of the very few that address the interdependency in decision‐making events and that provide some understanding of the micro‐structural processes that contribute to decision making.^20^ Our findings illustrate that ‘decision‐making practices are simultaneously retrospective, current and prospective in orientation’ (p. 438) within a temporal, spatial and organisational unfolding of the patient's journey of care shaped through the imaging investigation process.^18^ Each point of contact consisted of a decision‐making event made up of discrete decision‐making moments. Each decision‐making moment was informed by retrospective, current and/or prospective information and knowledge. These decision‐making moments are combined into clusters of temporal decisions formed within a decision event (see Fig. 1).

Through these events each associated point of contact follows a particular sequence, with the information distributed and integrated on the basis of the provider's role and expertise. Different groups of health care professionals contribute their own clinical knowledge and pieces of information to the decision‐making events.^21^ The result is that different health care providers with access to different pieces of information may have a different understanding and/or interpretation of the patient's situation,^22^ which in turn may influence the quality of patient‐centred care and the ultimate treatment decision.

This study also underlines the fact that decisions about patient care are not made in isolation, but are underpinned by a ‘distributed partnership’ (p. 67)^1^ between different health care providers, as well as between health care providers and patients. The interdependency between these provider partnerships is complemented by the mediating role of text/documents (e.g. request forms, medical files and radiologic reports) and technology (e.g. imaging equipment and the radiological information system), which could further influence treatment decisions.^23^ Thus, an appreciation and understanding of diagnostic imaging decision making could be described as a complex and dynamic system of activities in which negotiation is the key to an integrated team‐based decision‐making process. Role players could come to a shared understanding of the meaning of the processes and procedures in diagnostic imaging investigations through a deeper understanding of the transactions, interactions and processes of decision making. This illustrates the importance of health care providers engaging with the notion of ‘distributed knowing’^9^ in order to come to the best possible decisions regarding patient diagnosis and treatment.

This study had a number of limitations. The focus of the analysis was on the types of knowledge providers needed for imaging and clinical decision making, with less attention to the effect of teamwork and inter‐professional meetings.^9^ The study was, furthermore, only conducted in a single public health complex and aspects of decision making in action may look differently in other public settings or in the private sector.

The limited communication between patients and providers in a resource‐constrained setting did not allow for sufficient insights into the potential of shared decision making associated with patient‐centred care.^3^ The shadowing methodology did not appear to have influenced provider willingness to participate. Only in three instances did a medical practitioner decline audio recording or observation of a consultation, but all three were willing to share the process followed during the consultation process afterwards in an interview. The shadowing also appeared to have a positive effect on patient participants, as they had the opportunity to share their expectations and experiences in the entry and exit interviews, respectively, and their concerns in the informal interactions in‐between.

## Conclusion

This paper focused on distributed decision making in the context of diagnostic imaging investigations within a medical encounter. It illustrated the ‘when’ and ‘where’ of vital contact points between individual medical and non‐medical health care providers and between providers and patients. However, the distributed nature of decisions also illustrated how difficult it is to achieve the care traditionally associated with patient‐centred care. This can be attributed to the fragmented contributions of providers, who each come with an own interpretation of the retrospective, current and prospective knowledge at his or her disposal when making decisions. Because of radiation safety issues, it is proposed that a provider–patient‐centred approach mediated through and by technology may be a more appropriate perspective.^16^ According to Epstein et al., the locus of responsibility for shared decision making rests with the physician.^24^

Further investigation of interaction patterns (the ‘how’) that influence the nature and quality of the distributed decisions may be able to provide more insight into how to involve patients in decision making where diagnostic imaging investigations are conducted. This paper only explored decisions by individual practitioners within a team, based on single patient encounters with the health care system. Decision making through multiple encounters with the diagnostic imaging services by the same patient(s) also deserves further attention. Other levels of decision making that need further exploration relate to team decisions within an organisation and the ways in which organisational interactions influence decision making within the broader health care system.^25^

## Conflict of Interests

The authors declare no conflicts of interest.
